# Resilience of a FIT screening programme against screening fatigue: a modelling study

**DOI:** 10.1186/s12889-016-3667-8

**Published:** 2016-09-22

**Authors:** Marjolein J. E. Greuter, Johannes Berkhof, Karen Canfell, Jie-Bin Lew, Evelien Dekker, Veerle M. H. Coupé

**Affiliations:** 1 Department of Epidemiology and Biostatistics, VU University Medical Center, PO Box 7057, MF F-wing, 1007 MB Amsterdam, The Netherlands; 2 Cancer Research Division, Cancer Council NSW, Sydney, NSW Australia; 3 School of Public Health, Sydney Medical School, The University of Sydney, Sydney, NSW Australia; 4 Prince of Wales Clinical School, Faculty of Medicine, The University of New South Wales, Sydney, NSW Australia; 5 Department of Gastroenterology and Hepatology, Academic Medical Centre, Amsterdam, The Netherlands

**Keywords:** Colorectal cancer, Screening, Participation

## Abstract

**Background:**

Repeated participation is important in faecal immunochemical testing (FIT) screening for colorectal cancer (CRC). However, a large number of screening invitations over time may lead to screening fatigue and consequently, decreased participation rates. We evaluated the impact of screening fatigue on overall screening programme effectiveness.

**Methods:**

Using the ASCCA model, we simulated the Dutch CRC screening programme consisting of biennial FIT screening in individuals aged 55–75. We studied the resilience of the programme against heterogeneity in screening attendance and decrease in participation rate due to screening fatigue. Outcomes were reductions in CRC incidence and mortality compared to no screening.

**Results:**

Assuming a homogenous 63 % participation, i.e., each round each individual was equally likely to attend screening, 30 years of screening reduced CRC incidence and mortality by 39 and 53 %, respectively, compared to no screening. When assuming clustered participation, i.e., three subgroups of individuals with a high (95 %), moderate (65 %) and low (5 %) participation rate, screening was less effective; reductions were 33 % for CRC incidence and 43 % for CRC mortality. Screening fatigue considerably reduced screening effectiveness; if individuals refrained from screening after three negative screens, model-predicted incidence reductions decreased to 25 and 18 % under homogenous and clustered participation, respectively. Figures were 34 and 25 % for mortality reduction.

**Conclusions:**

Screening will substantially decrease CRC incidence and mortality. However, screening effectiveness can be seriously compromised if screening fatigue occurs. This warrants careful monitoring of individual screening behaviour and consideration of targeted invitation systems in individuals who have (repeatedly) missed screening rounds.

## Background

Several countries have implemented a colorectal cancer (CRC) screening programme based on faecal immunochemical testing (FIT) [1]. Due to imperfect sensitivity of FIT, around 20 % of CRCs and 70 % of advanced adenomas are missed in a single screening round [2–5]. To increase the probability of presymptomatic detection of advanced neoplasia, repeated screening is required. Thus, participation in multiple screening rounds is essential to underpin the effectiveness of FIT-based screening programmes to prevent CRC.

Only three studies have evaluated participation over multiple rounds of FIT screening [6–8]. These studies showed varying participation patterns; two Dutch studies concerning three screening rounds reported that participation increased in the third round [6, 7] whereas an Italian study concerning four screening rounds demonstrated a fluctuating participation pattern [8]. Regarding repeated participation in these studies, the majority of invitees participated at least once. More importantly, in one of the Dutch studies 54 % of invitees participated in all three rounds [7] whereas the Italian study reported a four round participation rate of 38 % [8].

Based on the limited and inconclusive data on participation, it is uncertain whether invitees of an 11-round FIT screening programme will stay motivated to attend screening. Invitees may lose the motivation to participate because of a false perception of decreased CRC risk after several negative test outcomes [9]. We denote this phenomenon by the term ‘screening fatigue’, leading to decreased participation. Screening fatigue may be a potential threat for FIT screening programmes since repeated testing is important to achieve reasonable sensitivity for advanced neoplasia. Furthermore, CRC risk increases with age [10] which stresses the importance of participation among older individuals.

We evaluated the potential impact of screening fatigue on long-term screening effectiveness in terms of CRC incidence and mortality reductions compared to no screening. We considered several scenarios differing in participation pattern, number of negative screens after which screening fatigue occurs and decrease in participation rate due to screening fatigue.

## Methods

### ASCCA model

The Adenoma and Serrated pathway to Colorectal CAncer (ASCCA) model, which is extensively described elsewhere, [11] was used for all analyses. This model simulates individual health trajectories from age 20 to age 90 or death, whichever comes first. During his lifetime, an individual can develop up to ten adenomas and ten serrated lesions. The development of each lesion in terms of growth in size and malignant features, i.e., dysplasia and villosity, is modelled independently. Only advanced adenomas can progress to CRC. Once a tumour has developed, there is each year a chance that the tumour becomes detected by symptoms, or progresses to a more advanced stage.

For the current evaluation, we assumed that all CRCs arise from adenomas to allow for comparability of model results to other CRC models. Thus, serrated lesions are considered innocuous. The model satisfactorily reproduces the Dutch sex- and age-specific adenoma prevalence, as well as Dutch CRC incidence and mortality rates [11].

### Screening programme

The model was set up to simulate the Dutch CRC screening programme consisting of biennial FIT screening. Phased implementation of this programme has started in 2014. Each year, more birth cohorts are included until full implementation in 2019. From that year onwards, all individuals aged 55 to 75 will be biennially invited. Because data from the fully implemented programme are not yet available, we derived the participation rate from a pilot study in which participation was 63 % [7].

A positive test is followed by referral to diagnostic colonoscopy. We set adherence to diagnostic colonoscopy at 83 %, as observed in the Dutch screening programme in 2014 [12]. Of note, a Dutch screening implementation trial reported similar figures for all age groups [6]. During colonoscopy, all detected adenomas are completely removed. Furthermore, we incorporated a small risk of complications to include both screening benefit and burden. Colonoscopy surveillance is modelled in accordance with Dutch guidelines [13]. That is, a risk score is calculated based on the findings during colonoscopy. This risk score determines the surveillance interval, i.e., 3 or 5 years. Similarly to diagnostic colonoscopy, participation for surveillance was set at 83 %. Surveillance ends at age 75.

### Test characteristics

Table 1 shows test characteristics of FIT and colonoscopy. FIT sensitivity and specificity were obtained by calibration, following a previously reported procedure [11]. With these characteristics we were able to reproduce positivity rates, detection rates and positive predictive values as reported in a Dutch FIT screening trial [14]. Detection rates of colonoscopy were based on a systematic review on polyp miss rates [15].

**Table 1:** Test characteristics of FIT and colonoscopy

Variable	Value		Reference
FIT characteristics per lesion	Men	Women	[11, 14]
*Specificity*	0.96^a^	0.97^a^	
*Sensitivity diminutive adenoma*	0.0041	0.003	
*Sensitivity small adenoma*	0.12	0.10	
*Sensitivity large adenoma*	0.30	0.28	
*Sensitivity CRC early stage*	0.50	0.50	
*Sensitivity CRC late stage*	0.85	0.85	
Colonoscopy miss rate			[15]
*Diminutive adenoma*	0.26		
*Small adenoma*	0.13		
*Large adenoma*	0.021		

^a^Specificity per person

### Scenarios

Besides the comparator scenario without screening, we considered sixty-two screening scenarios differing in participation pattern, number of negative screens after which screening fatigue occurs and decrease in participation rate due to screening fatigue. We considered two participation patterns, in order to assess the effect of repeated participation. In the first pattern, each individual has each round the same probability, i.e., 63 %, of participating in screening. Thus, participation in a single round is not correlated to participation in previous rounds. We refer to this pattern as homogenous participation.

The second participation pattern is described as clustered participation and is based on the observation that some individuals participate in most screening rounds whereas others participate only sporadically [7, 8, 16]. Individuals in the model were allocated to a high, moderate or low participation group, in which each group was assigned a different participation rate, i.e., 95, 65 and 5 % respectively. To determine the percentage of individuals in each subgroup, the model was calibrated against data from a Dutch study concerning three rounds of FIT screening [7]. Calibration targets included a 63 % overall participation rate per round, the percentage of individuals who completed all screening rounds and the percentage of individuals who participated at least once. Respectively 45, 25 and 30 % of the population were allocated to the high, moderate and low participation subgroup. We assumed that individuals remain in the same participation group during the entire screening programme.

We first simulated two scenarios differing in participation pattern in which screening fatigue did not occur. Subsequently, to fully evaluate the potential threat of screening fatigue, we hypothesized that it could occur after any specific number x of negative screens, with x running from one to ten. We did not assume that negative screens were necessarily consecutive because individuals do not participate in every round due to imperfect participation. Furthermore, after onset of screening fatigue at screen x, we assumed a decrease in participation of either 25 or 50 % (relative decrease) or that individuals would refrain from screening. This resulted in 60 screening scenarios in which screening fatigue occurred.

### Analyses

We modelled screening from 2014 to 2044 while accounting for phased implementation. We started with a population based on the 2013 Dutch population age-composition and assumed that this population will age in accordance with predictions of the Central Bureau of Statistics [17]. Outcomes of each strategy were model-predicted CRC incidence and mortality per 100,000 individuals per year.

Furthermore, we calculated individual CRC risk after any number of consecutive negative screens, if the individual would refrain from further screening. For this purpose, we simulated a cohort of 20,000,000 individuals assuming that all individuals are fully compliant with screening. Screening was ceased after one to eleven screens and individuals with a positive FIT at any round were excluded. Outcomes were age-specific CRC incidence and mortality per 100,000 individuals as well as CRC risk in the remaining life-time of a 60-, 64-, 68-, 72- and 76-year old with consecutive negative screens.

Since this study focused on evaluation of multiple behavioural scenarios, detailed sensitivity analysis for any particular scenario was not performed. Rather, the sensitivity of outcomes were characterised via the multiple screening scenarios examined.

## Results

### Impact of screening

In the scenario without screening, model-predicted age-standardised CRC incidence and mortality rates were respectively 77 and 30 per 100,000 individuals in 2014. Due to population ageing, these rates increased to 109 and 44 per 100,000 individuals, respectively, in 2044. Thirty years of screening significantly decreased both CRC incidence and mortality. However, reductions were dependent on participation pattern. When assuming homogenous participation, CRC incidence and mortality rates in 2044 were respectively 67 and 21 per 100,000 individuals. These rates correspond to a reduction of 39 and 53 % compared to no screening. Screening impact under clustered participation was lower; CRC incidence decreased by 33 % whereas CRC mortality decreased by 43 % compared to no screening.

Assuming homogenous participation, individuals participated on average in six screening rounds [5th - 90th percentile: 4–9]. For clustered participation, the average number of rounds was five. When considering the different participation groups separately, individuals in the high, moderate and low participation group attended on average eight [5th - 95th percentile: 9–11], six [5th - 95th percentile: 4–10] and one [5th - 95th percentile: 0–2] screening round, respectively. These figures include individuals who do no longer participate due to a positive test result and thus, are referred to diagnostic colonoscopy and surveillance.

### Impact of screening fatigue

**Fig. 1:**
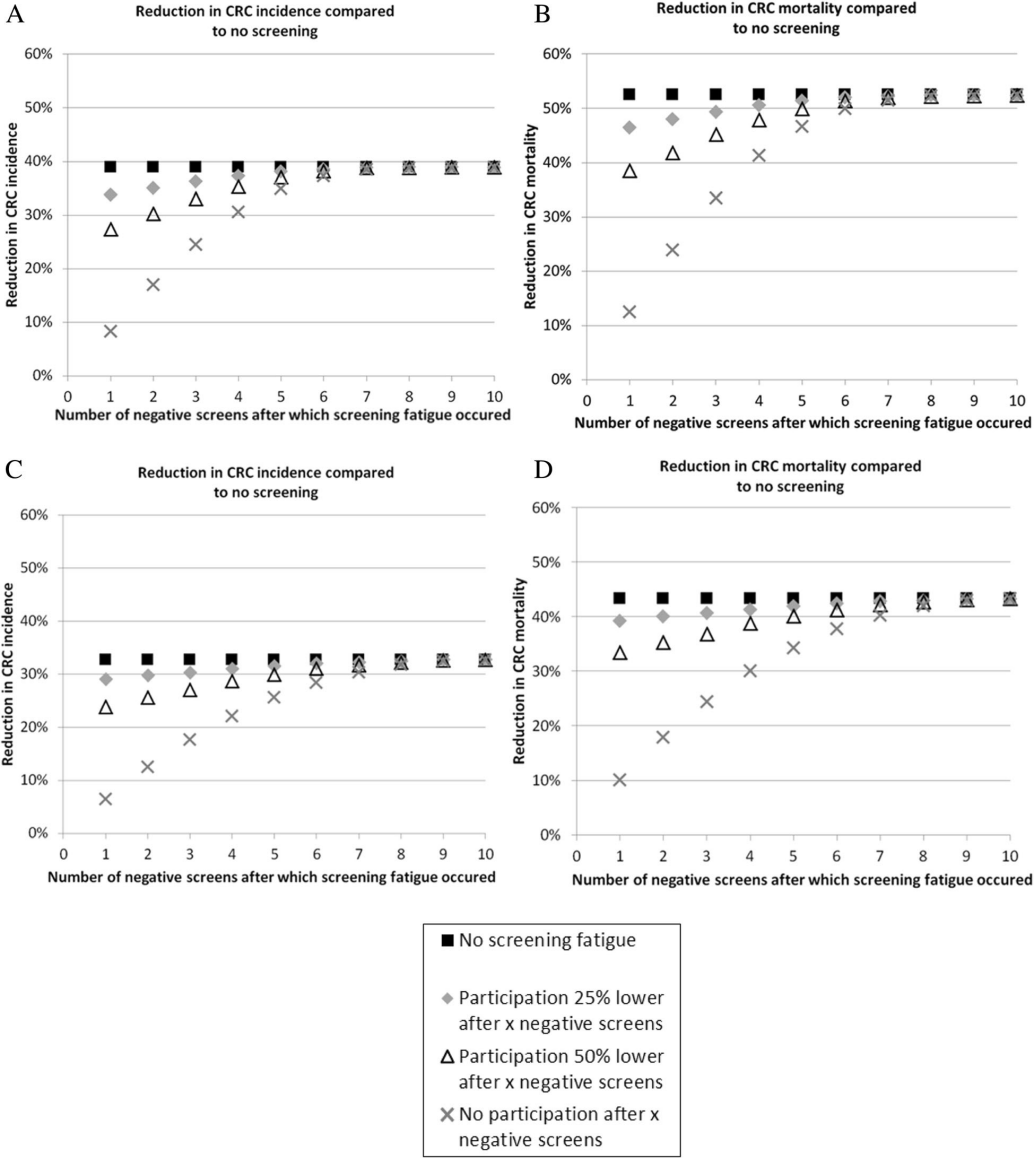
Reduction in CRC incidence (**a**) and mortality (**b**) assuming homogenous participation and reduction in CRC incidence (**c**) and mortality (**d**) assuming clustered participation after thirty years of population-based screening compared to no screening for different scenarios of screening fatigue

We evaluated the impact of screening fatigue assuming both homogenous and clustered participation. Figure 1 shows reductions in CRC incidence and mortality compared to no screening for different scenarios of screening fatigue assuming homogenous participation. As expected, screening fatigue had the highest impact on screening effectiveness when it occurred after the first negative screen and individuals would no longer participate from that point, since this minimised total lifetime exposure to screening. In that case, screening effectiveness decreased from a 39 % (no screening fatigue) to an 8 % reduction in CRC incidence and from a 53 % (no screening fatigue) to a 13 % reduction in CRC mortality.

In the situation where screening fatigue did not occur before six negative screens, and the decrease in participation was limited to 25 %, the long-term impact of screening was not substantially affected. However, if screening fatigue occurred after one to five negative screens, particularly if participation in subsequent rounds decreased by 50 % or even 100 %, the long-term impact of screening was more substantially attenuated. For example, if individuals refrained from further screening after three negative screens, reductions in CRC incidence and mortality were respectively 25 and 34 %.

Figure 1 shows reductions in CRC incidence and mortality compared to no screening, assuming clustered participation. The observed pattern was roughly similar as for homogenous participation, but the impact of screening fatigue seems to be slightly more pronounced. For example, if individuals refrained from further screening after three negative screens, reductions in CRC incidence and mortality were respectively 18 and 25 %.

### CRC risk after consecutive negative screens

**Fig. 2:**
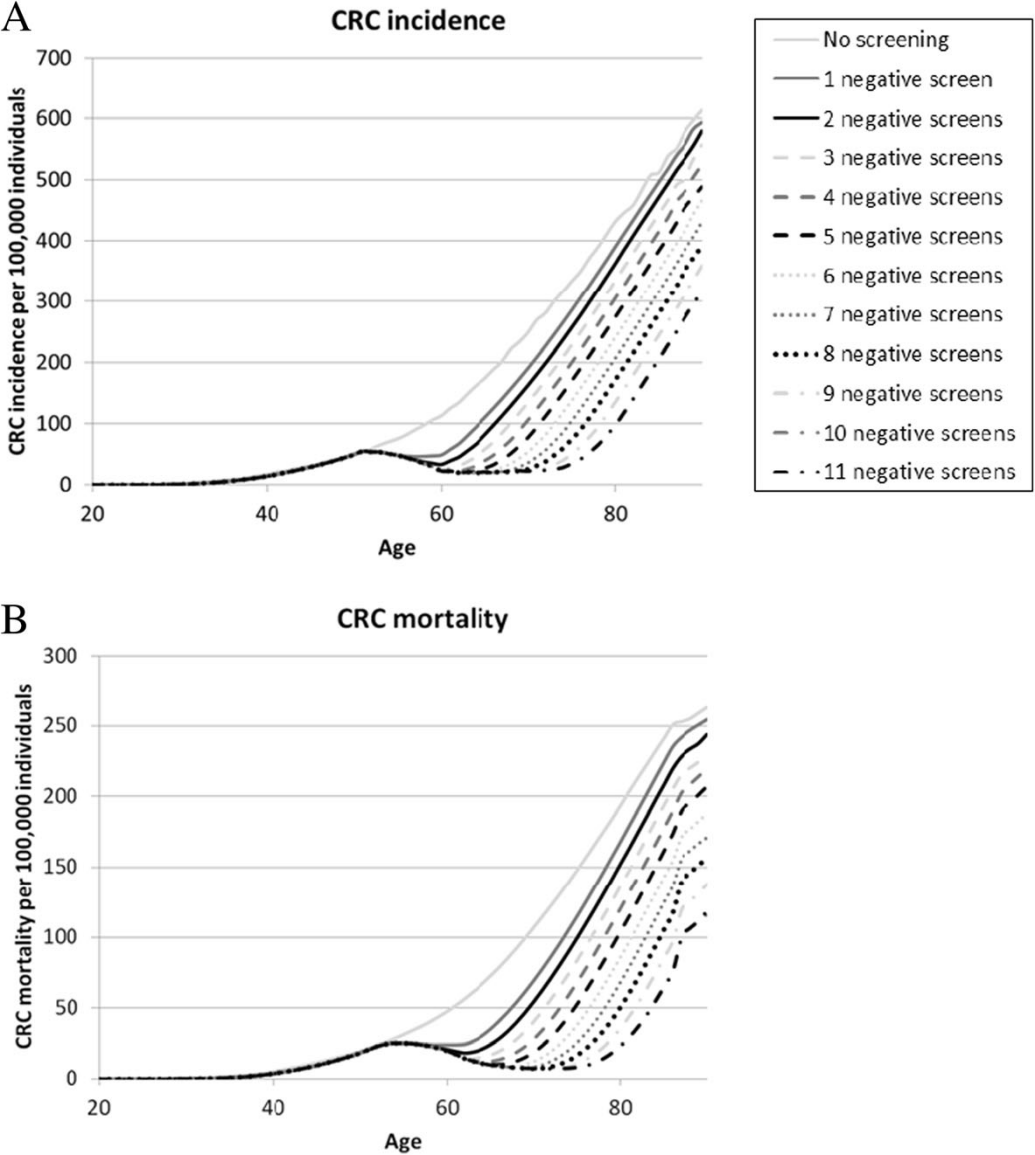
Age-specific CRC incidence (**a**) and mortality (**b**) after one to eleven consecutive negative screens

Figure 2 shows age-specific CRC incidence and mortality in individuals who had one to eleven consecutive negative screens as well as in never-screened individuals. Individuals with one or more negative screens had a lower CRC risk than never-screened individuals. For example, model-predicted CRC incidence at age 75 was 334/100,000 in never-screened individuals. The incidence decreased to 284/100,000 in individuals who had one negative screen at age 55 years and to 34/100,000 in individuals who had eleven negative screens between age 55–75. Each additional negative screen decreased CRC risk, except for the eleventh negative screen; CRC incidence was comparable in individuals who had ten or eleven negative screens. For CRC mortality, a similar pattern was observed.

Table 2 shows CRC risk in the remaining lifetime of a 60- to 76-year old individual after one to eleven consecutive negative screens. In never-screened individuals, the predicted lifetime risk varied between 6.3 % for a 60-year old to 4.4 % for a 76-year old. Each negative screen lowered the lifetime risk, except for the eleventh negative screen.

**Table 2:** Risk of CRC in the remaining lifetime of a 60-, 64-, 68-, 72- and 76-year old individual after one to eleven consecutive negative screens

Number of consecutive negative screens	Lifetime risk of CRC (%) in a				
	60-year old	64-year old	68-year old	72-year old	76-year old
0^a^	6.3	6.0	5.6	5.1	4.4
1	5.2	5.2	5.0	4.6	4.1
2	4.7	4.7	4.6	4.3	3.9
3	3.9	4.2	4.2	4.0	3.6
4	^b^	3.7	3.7	3.6	3.3
5	^b^	3.0	3.3	3.2	3.0
6	^b^	^b^	2.8	2.8	2.7
7	^b^	^b^	2.2	2.4	2.4
8	^b^	^b^	^b^	2.0	2.0
9	^b^	^b^	^b^	1.5	1.7
10	^b^	^b^	^b^	^b^	1.3
11	^b^	^b^	^b^	^b^	1.3

^a^No screening scenario

^b^We assumed the individual would refrain from further screening

## Discussion

This study assessed the impact of screening fatigue on long-term screening effectiveness. When assuming homogenous participation, 30 years of screening led to a 39 and 53 % model-predicted reduction in respectively CRC incidence and mortality. Screening was less effective when assuming clustered participation; reductions were 33 and 43 %. Screening fatigue considerably reduced screening effectiveness. If individuals refrained from further screening after three negative screens, reductions in CRC incidence were decreased to 25 and 18 % under homogenous and clustered participation, respectively. Figures were 34 and 25 % for mortality reduction.

In all scenarios, screening effectiveness was lower assuming clustered participation compared to homogenous participation. This holds for screening in general. However, the extent of the difference in screening effectiveness is dependent on the particular clustering pattern. Since we based our assumptions for clustering on a Dutch study only [7], other clustering patterns may also occur which will affect the difference in screening effectiveness.

In clustered participation, which is probably closer to reality than homogenous participation, more individuals are subjected to multiple screening rounds whereas in homogenous participation, more individuals are subjected to at least one screening round. This suggests that efforts to increase participation in non-attendees may be more beneficial to enhance screening effectiveness than attempts to increase repeated participation. Studies evaluating participation over multiple rounds of FIT screening have shown that the group of non-attendees is considerable; in two screening rounds, 25 to 35 % of invitees will not participate [7, 16] and this is still around 25 % in three or four screening rounds [7, 8]. Participation can be increased by, for example, advance notification letters and reminders [18].

Assumptions about participation behaviour vary widely among CRC screening models. For example, the SimCRC model assumes that 10 % of individuals are never screened, whereas the remaining 90 % are equally divided among a low, moderate and high participation group [19]. The MISCAN model also assumes that 10 % of individuals are non-attendees but does not allocate individuals to subgroups differing in participation. However, they incorporated correlated participation by assuming that 80 % of individuals that participated in a certain round will also participate in the next round [20]. These differences in assumptions will lead to differences in model predictions. To improve the accuracy of model-based predictions of screening impact, access to data on individual screening behaviour is extremely valuable.

For homogenous and clustered participation, long-term screening effectiveness was hardly affected by screening fatigue when it occurred after respectively seven or nine negative screens. This is due to the fact that few individuals reach that number of negative screens. Under homogenous participation, each individual had a 63 % chance to participate in screening every 2 years. Of the participating individuals, around 6 % has a positive FIT [21]. Furthermore, individuals in the screening eligible age range are at risk of dying of other causes then CRC. Consequently, few individuals reach seven or more negative screens. Under clustered participation, slightly more individuals will reach a high number of negative screens due to the 95 % participation rate in the high participation group (45 % of population). Therefore, the potential impact of screening fatigue is higher assuming clustered participation. Note that this does not imply that FIT screening programmes could actually be halted earlier, that is, after seven (homogenous participation) or nine (clustered participation) screening rounds. It is due to imperfect participation that as many as eleven rounds should be offered to achieve on average six (homogenous participation) or five (clustered participation) rounds. Moreover, our analyses of CRC risk in the remaining lifetime after one to ten negative screens showed that for an individual, every additional negative screen led to a further decrease in CRC risk. Note that even after eleven negative screens, CRC risk is still non-negligible.

In this study, we have focused on the effectiveness of continued screening in individuals with multiple negative screening tests. Another viewpoint that could be taken is that of the cost-effectiveness of continuing to offer biennial screening in individuals with multiple negative screens; this may not be a cost-effective strategy. In cervical cancer screening, studies have reported that several consecutive negative tests imply reduced risk [22–25]. This has led, in some settings, to current guidelines for less frequent screening after multiple negative cervical cancer screening tests, [26–28] although this is not a usual feature of most screening programmes. It is possible that less frequent CRC screening may in the future be justified in individuals with multiple consecutive negative tests, but a comprehensive evidence base including observational data from screening programmes would be required on the long-term protective effects, in order to support such a recommendation. Further cost-effectiveness research, grounded in such evidence, would also be required. On the other hand, such an individualized screening strategy is complex to organize. Moreover, with respect to FIT screening, there are still important issues to address first such as optimizing currently suboptimal participation rates.

Three studies have investigated participation in FIT screening in multiple screening rounds [6–8]. It is impossible to extrapolate participation behaviour in these studies to a full programme consisting of eleven screening rounds because results are inconsistent and the number of rounds studied is still limited. For example, Stegeman et al. [6] reported a slight increase in participation rate in the third round. This increase was more pronounced in a comparable Dutch study [7]. In contrast, participation rates in an Italian study evaluating four rounds were fluctuating; participation was around 56 % in the first and third round and around 62 % in the second and fourth round [8].

Data on participation in more than four FIT screening rounds is not available. Also individual participation patterns are not reported. Participation is likely to be setting-specific and depends on several factors including the organisation of screening and associated communication and health promotion activities. Therefore, it is still unknown if screening fatigue will occur in a particular setting. We showed that screening fatigue can have a considerable impact on screening effectiveness. This warrants careful monitoring of individual participation behaviour in FIT screening.

In a programme consisting of only a few screening rounds, the probability that screening fatigue will occur is likely to be small. Therefore, the use of a highly sensitive screening test is important because this enables elongation of the screening interval. For example, in colonoscopy screening, the interval between subsequent rounds can be safely extended to 10 years [29] and only a small number of screening rounds in a lifetime is required.

Our study has several limitations. Firstly, individuals with an increased CRC risk, e.g., men and low SES individuals, [30–32] might be more prone to screening fatigue since they are already less likely to participate in screening [33]. We did not take this into account.

Furthermore, we assumed that screening fatigue would occur after the same number of negative screens in all individuals whereas it is more likely that this will differ between individuals. However, this variation is implicitly included in the scenarios where we assumed a decrease in participation due to screening fatigue; individuals still have a chance of participating in subsequent screening rounds.

We also did not differentiate between individuals who had x consecutive negative tests and individuals who had x negative tests with a longer time-period in between. We believe this would not have substantially affected our results, because there is considerable variation in individual participation patterns due to imperfect compliance.

On the other hand, we simulated a realistic screening programme, assuming participation rates based on screening pilots and accounting for phased implementation. Furthermore, we evaluated numerous strategies differing in participation pattern, number of negative screens after which screening fatigue occurs and decrease in participation rate due to screening fatigue, in order to comprehensively evaluate the potential impact of screening fatigue.

## Conclusion

Screening will considerably decrease CRC incidence and mortality. However, the effectiveness of a FIT screening programme can be seriously compromised if screening fatigue occurs. This warrants careful monitoring of individual screening behaviour and consideration of targeted invitation and reminder systems for screening in individuals who have (repeatedly) missed screening rounds.

## Abbreviations

ASCCA: Adenoma and Serrated pathway to Colorectal CAncer
CRC: colorectal cancer
FIT: faecal immunochemical testing
